# Intraoperative radiation safety in orthopaedics: a review of the ALARA (As low as reasonably achievable) principle

**DOI:** 10.1186/s13037-016-0115-8

**Published:** 2016-12-12

**Authors:** Daniel J. Kaplan, Jay N. Patel, Frank A. Liporace, Richard S. Yoon

**Affiliations:** 1 Department of Orthopaedic Surgery, RWJBarnabas Health - Jersey City Medical Center, 355 Grand St, Jersey City, NJ 07302 USA; 2 Division of Orthopaedic Traumatology & Complex Adult Reconstruction, Department of Orthopaedic Surgery, Orlando Regional Medical Center, 1222 S Orange Ave, 5th Floor, Orlando, FL 32806 USA

**Keywords:** Orthopaedic surgery, Radiation safety, Radiation exposure, Radiation, Operating room safety, Fluoroscopy, c arm, Surgical training

## Abstract

The use of fluoroscopy has become commonplace in many orthopaedic surgery procedures. The benefits of fluoroscopy are not without risk of radiation to patient, surgeon, and operating room staff. There is a paucity of knowledge by the average orthopaedic resident in terms proper usage and safety. Personal protective equipment, proper positioning, effective communication with the radiology technician are just of few of the ways outlined in this article to decrease the amount of radiation exposure in the operating room. This knowledge ensures that the amount of radiation exposure is as low as reasonably achievable. Currently, in the United States, guidelines for teaching radiation safety in orthopaedic surgery residency training is non-existent. In Europe, studies have also exhibited a lack of standardized teaching on the basics of radiation safety in the operating room. This review article will outline the basics of fluoroscopy and educate the reader on how to safe fluoroscopic image utilization.

## Background

One of the most valuable tools in an orthopaedic surgeon’s armamentarium is the fluoroscopic imaging (c-arm) unit. Although fluoroscopy is utilized on a daily basis, there is a paucity of knowledge by the average orthopaedic trainee in terms proper usage and safety. By learning the basics of how a c-arm operates, one may better understand how to obtain useful images. Effective communication with the technician allows efficient acquisition of images with decreased risk to the patient and staff. Currently, there are no universally accepted guidelines for minimizing radiation exposure in the operating room. Furthermore, there is no standardized curriculum in orthopaedic residency training in teaching radiation safety. Many training sites have no orthopaedic training in radiation safety. This review article will outline the basics of fluoroscopy and educate on how to best utilize this tool.

### Current protocols for intraoperative radiation safety in orthopaedic training

Radiation safety and proper c-arm use instruction varies greatly from residency training site to residency training site. A recent survey of Irish Orthopaedic trainees demonstrated low compliance with several important techniques in reducing radiation exposure. Only 65% of trainees reported attending a radiation safety course at some point in their training. 69% were aware of the As Low As Reasonably Achievable (ALARA) principle to reduce radiation exposure. 96% of respondents used lead aprons, but a much lower percentage used thyroid shields or dosimeters. Surprisingly, 62% of respondents did not believe any additional protection was required in pregnancy. Common barriers to adherence to safety protocols included unavailability of protective equipment or the thought that the protocols were unnecessary [1].

A second study of basic surgical trainees also demonstrated a lack of knowledge and adherence to techniques in order to decrease radiation exposure. Only 18% of respondents reported reading any literature on radiation safety during their training. 24% reported using a thyroid shield [2]. These studies demonstrate a clear need for additional education in radiation safety for residents.

### Basics of radiation

**Fig. 1:**
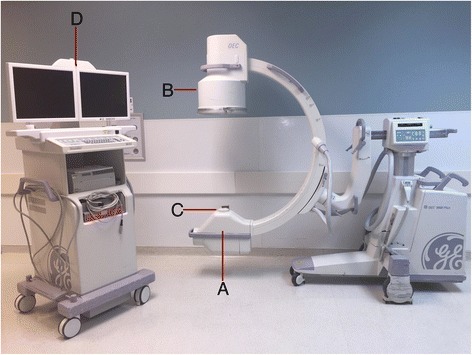
Basic c-arm unit. **a**. X-ray Tube; **b**. Image Intensifier; **c**. Collimator; **d**. Display Monitor

A fluoroscopic unit consists of an electron source, an evacuated tube, a target electrode and an external power source. A cathode acts as the source of electrons, while the anode is the target of the electrons. The external power source creates an electrical potential difference within a vacuum and is responsible for the acceleration of electrodes as they travel from the cathode to the anode. X-rays are created by the interaction of electrons with matter, with conversion of some of their kinetic energy into electromagnetic radiation [3]. Figure 1 diagrams the basic parts of a c-arm unit. The function of each part is outlined in Table 1.

**Table 1:** Basic parts of the c-arm unit

C-arm Part	Function
X-ray Tube	Source of x-ray beam
Image Intensifier	Captures the x-ray beams and converts them into an image that is displayed on the display monitor
Collimator	Contains various apertures that determine the size and shape of the x-ray beam
Display Monitor	Displays the x-ray

The x-rays interact with bone, soft tissue, and air within the patient resulting in different patterns of x-ray distribution. X-rays that pass through the patient and reach the x-ray detector, result in formation of a radiographic image. X-rays that are not absorbed are deflected and continue on with lower energy [4]. This pattern of deflection, or scatter, produces a field of radiation that is responsible for the incidental radiation exposure to the surrounding staff [5].

There are several units of measurement that need to be understood when describing radiation exposure. The units Gray (Gy) and Rad are used to measure the absorbed dose, that is the amount of physical energy that is deposited in matter. One Gy equals 1 Joule per Kg of matter. One Gy equals 100 Rads. The units Sievert (Sv) and Roentgen equivalent man (Rem) are used to measure the equivalent dose. The equivalent dose is used to estimate the biological damage from the various types of radiation that is absorbed by tissues. One Sv is equal to 100 Rem. A given dose of radiation will have different effects depending on the type of radiation and the type of tissues affected. To determine the equivalent dose (Sv), you multiply the absorbed dose (Gy) by a quality factor (Q) that is unique to each type of radiation.

### Effects of radiation on living tissue

Radiation damage occurs at the cellular level in living tissues. Rapidly replicating cell components such as DNA and cell membranes are the most susceptible to damage from radiation. This may occur by both direct and indirect mechanisms. Direct damage occurs as energy is absorbed and molecular bonds are broken. This can result in cell death or distorted replication, and is thought to be the initial step in radiation-induced carcinogenesis. Indirect damage occurs when water molecules are ionized into free radicals, which have the ability to disrupt bonds. Indirect action is thought to be responsible for the long-term effects of radiation [4].

Orthopaedic surgeons have been shown to have an increased incidence risk of cancer compared to non-exposed workers [6]. The thyroid, eyes, hands, and gonads are among the most sensitive organs to radiation exposure. The eyes may exhibit the first effects of chronic radiation exposure in the form of cataracts [7]. Eighty five percent of papillary carcinomas of the thyroid are thought to be radiation induced [8]. A surgeon’s hands have the greatest exposure risk due to their constant proximity to the radiation beam. Due to these risks, the International Commission on Radiological Protection established dosage limits for radiation exposure. The maximum annual dose limit is 20 mSv for the body, 150 mSv for the thyroid and eyes, and 500 mSv for the hands [9].

### Personal protective equipment

As fluoroscopic use has become more commonplace, it is imperative that an Orthopaedic surgeon becomes more familiar and comfortable with personal protective equipment (PPE). PPE contains lead or similar lightweight materials that attenuate scattered x-rays. There are multiple designs of PPE that may be worn by operating room personnel. Aprons may be one-piece front shielding or offer 360° coverage. Two-piece garments include an overlapping vest and skirt combination that may distribute weight better. These aprons are evaluated in lead-equivalent thickness. A lead-equivalent thickness of at least 0.5 mm is typically required, which attenuates over 95% of scattered x-rays that strike it [10].

Lead aprons must be inspected annually for damage that may cause x-rays to pass through. This may include cracks from improper folding or storage. Another important piece of PPE are thyroid shields. Thyroid shields are typically included in commercially available lead aprons. As noted earlier the thyroid is one of the most sensitive organs to radiation. Past studies have shown the protection offered by thyroid shields in the operating room during upper and lower extremity cases [10, 11]. In our anecdotal experience, thyroid shields can be the most difficult piece of PPE to find in the operating room often causing surgeons and staff to forgo their use. Aside from proper storage, both personal and shared thyroid shields should be cleaned after each use, as they may be an often overlooked source of possible infection [12].

Protective eyewear is commonly used in interventional radiology and is becoming more commonplace in Orthopaedics. The lens of the eye is a radiosensitive anatomic structure and must be protected from scatter. Leaded eyewear should include lateral protection, as the eyes are susceptible to backscatter from the head and direct scatter when the head is turned [13]. Leaded eyewear can reduce the exposure to the eyes by up to 90% in pelvic and hip surgery [14].

The hands have the greatest exposure to direct radiation during surgical procedures and are the most difficult to protect. The hand is often placed directly in the x-ray beam when positioning the operative extremity or surgical instruments for an x-ray. Gloves produce greater scatter and exposure to the hand within the glove from radiation that is not attenuated [15]. Sterile protective gloves are available, however they do not offer nearly as much protection as aprons or thyroid shields. Hands should not be placed directly in the beam when at all possible. The use of a Kocher forceps or other surgical tool to aid with positioning may help reduce exposure of the hands when obtaining images. Gloves cannot substitute for proper technique. Additional shields mounted on the table, ceiling, or on wheels should be utilized in the operating room whenever available.

### Scatter

In addition to reducing direct radiation exposure and wearing personal protective equipment, knowledge of the direction of scatter may further reduce exposure. The ALARA principle refers to reducing the amount of radiation delivered without compromising the integrity of imaging [16]. The benefit of obtaining imaging must exceed all risks to the patient and operating room personnel, including radiation exposure. Furthermore it is important to achieve the necessary diagnostic information with as little radiation exposure as possible. This principle should be kept in mind when using fluoroscopy in order to keep the patient, physician, and operating room team safe.

**Fig. 2:**
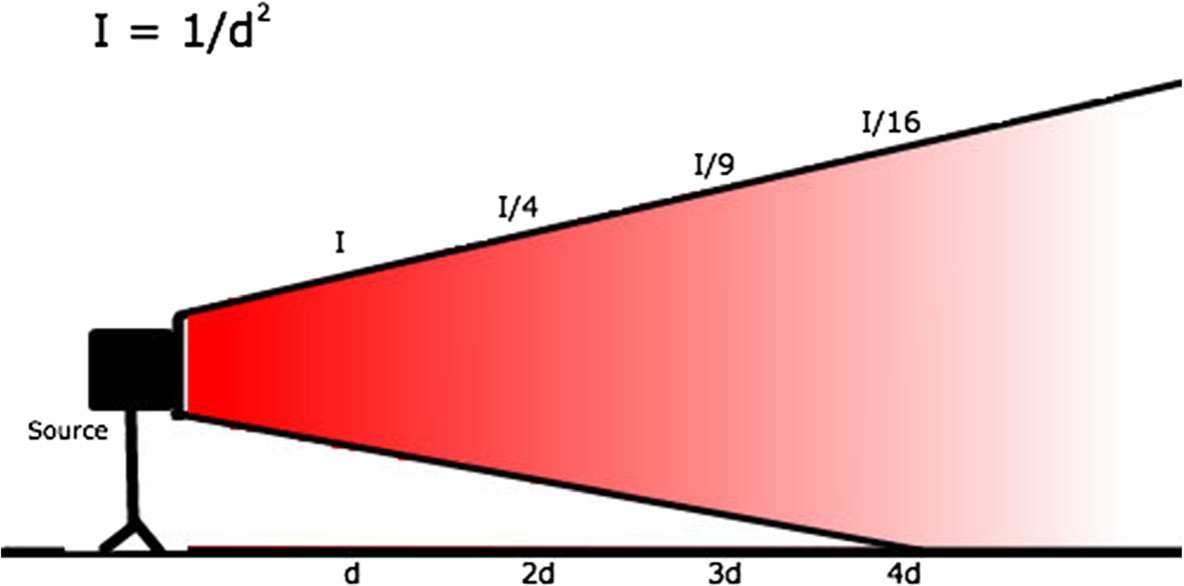
Inverse-square law: I = 1/d^2^, where I = magnitude of scatter and d = distance from the source. By doubling the distance from the x-ray tube, you receive only one fourth of the exposure from scatter

**Fig. 3:**
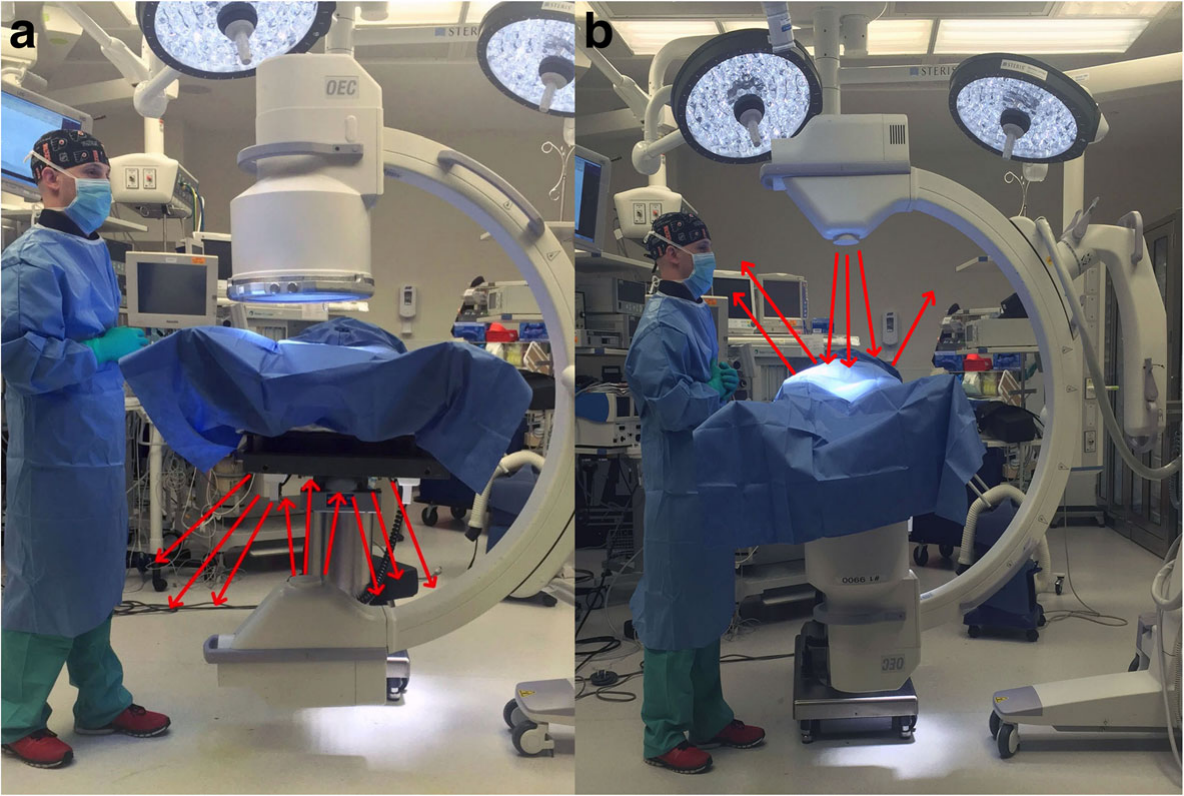
**a** shows a setup with the x-ray tube on the bottom. The red arrows represent radiation beams that scatter after they deflect off of the object being imaged. With the x-ray tube on the bottom most of the scattered (deflected) radiation is towards the legs and feet of the surgeon. **b** shows a setup with the x-ray tube on the top. Here the scattered radiation is towards the head and neck region of the surgeon

An understanding of the direction and magnitude of scatter can help reduce exposure. Scatter levels decrease proportionally to the inverse of the distance squared from the x-ray tube. This is known as the inverse-square law, intensity = 1/d^2^, where d = distance is from the source. By doubling the distance from the x-ray tube, you receive only one fourth of the exposure from scatter (Fig. 2). The highest rate of scatter is produced between the x-ray tube and the patient. This may produce higher scatter exposure levels at either the legs and feet or the head and neck of the surgeon depending on how the fluoroscopic unit is positioned as demonstrated in Fig. 3. Because of this, the x-ray tube is usually positioned underneath the patient. This relationship remains true when obtaining lateral views and should be considered when positioning the fluoroscopic unit. Standing on the opposite side of the table from the x-ray tube can greatly decrease scatter exposure when obtaining lateral c-arm imaging. With the inverse square law in mind, positioning of the image intensifier should be as close to the patient as possible. This can also be thought of as the source-to-extremity distance. Decreasing the distance between the image intensifier and the patient also increases the field of view captured in the x-ray.

As the thickness of an area being imaged increases, more x-ray beams will be required in order to achieve an image of similar quality. Therefore, as the size of a patient increases, the dose to the patient’s skin and amount of scatter increases as well. For this same reason a lateral image of the pelvis typically results in a higher dose delivered compared to an AP image of the same region. Magnification of the image also greatly increases the dose to both the patient and surgeon, and should be used only when necessary [17].

Continuous or live fluoroscopy allows the surgeon to image the surgical site in real-time and gain a better three-dimensional understanding. It may be useful when examining for perforation of screws into a joint in fracture care. Continuous imaging obtains about 30 images per second, which increases the amount of radiation exposure. Pulsed fluoroscopy obtains 1–6 images per second, which lowers the amount of radiation exposure [17].

**Fig. 4:**
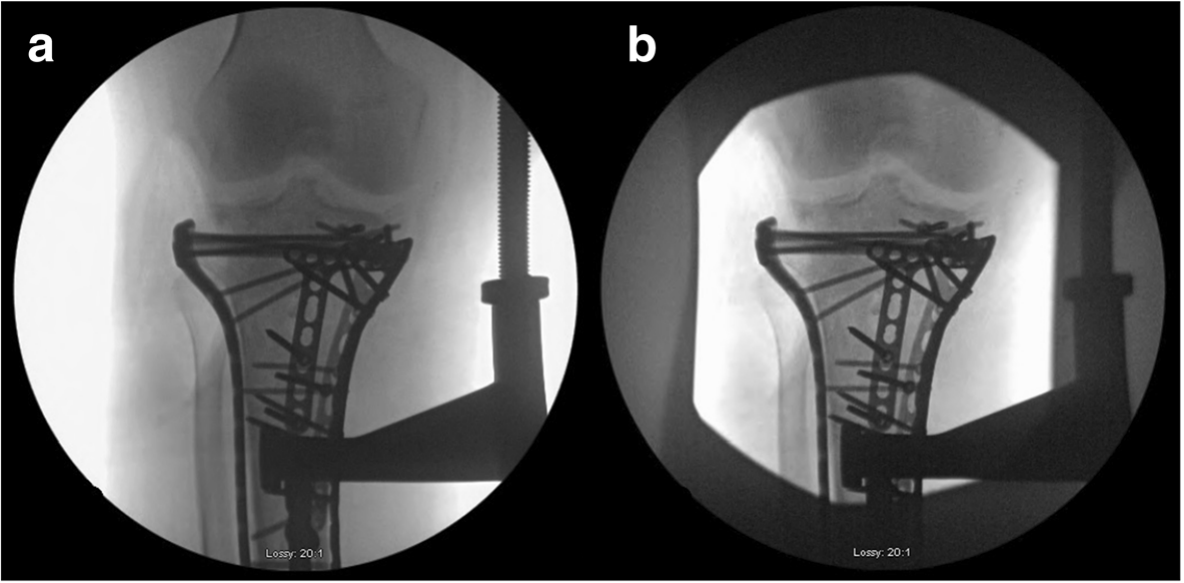
**a** shows a standard x-ray taken without collimation. **b** is an x-ray taken with collimation; collimation helps reduce exposure and may also help produce sharper images

Other ways to reduce exposure include laser targeting, landmarks, and manipulation of the x-ray beam. Collimation is performed by adjusting the size of the aperture that x-ray beams pass through when leaving the tube as seen in Fig. 4. This decreases the area of the direct x-ray beam and subsequently decreases the dose delivered and scatter [17]. Identifying and drawing anatomic landmarks on the patient or drapes can also assist the surgeon and technician in obtaining the needed imaging with decreased amounts of shots. Similarly, tape markers may be placed on the floor to assist the technician in returning the c-arm to its proper position when multiple projections are being obtained. Establishing these parameters prior to draping will allow the technician to move the c-arm to a predetermined position and decrease the amount of images shot and operative time. Laser targeting can further assist in decreasing exposure in a similar matter [18].

### Establishing proper terminology and communication

There is often a discrepancy in the dialogue used between radiology technicians and surgeons. An understanding of the terminology used and the ability to effectively communicate with the c-arm technician must be present in order to decrease exposure, reduce frustration and conflict, and avoid wasting valuable operating room time. The many movements a c-arm performs can result in increased confusion on how to best direct technicians. Many radiology technicians do not spend all of their time in the operating room, and work with multiple surgeons who use different terminology. All of these factors set up a perfect storm for inefficient communication and unnecessary radiation exposure.

Members of the Canadian Orthopaedic Association received 12 images that demonstrated each of the c-arm’s movements and were asked to describe how they would instruct a technician to perform these maneuvers. Surgeons were also asked how they would ask for a single image vs. live fluoroscopy. A great deal of diversity in the terminology used for specific movements was found. Furthermore, many ambiguous words such as “up”, “rotate”, or “turn” were used to represent the same movement. In the second part of the study, the most frequently used terms were used to create a multiple-choice test and given to members of the Canadian Association of Medical Radiation technicians. The authors then proposed a system of terms based on both parties responses. For linear movements it was suggested that each command consist of a direction, followed by a distance. For rotational movements a term describing the motion should be followed by a direction and magnitude in degrees [19].

Yeo et al [20] further illustrated the importance of a pre-arranged communication system between surgeons and technicians. 15 pairs of surgeons and technicians were evaluated in overall time and number of images required to obtain perfect circles before and after clear terminology was established. Perfect circles were simulated using a basketball with two washers taped to each side. All parameters significantly improved after the pair established effective consistent terminology. Time taken to establish perfect circles decreased from an average of 212–97 s, while the number of images taken decreased from 12 to 6. Improvements were more significant in pairs in which one of the member’s primary language was not English. Average time to establish an understanding of the new terminology was 109 s. Taking time prior to incision to establish common communication can save valuable time, improve teamwork, and decrease radiation exposure.

### Mini C-arm

The use of the mini c-arm has increased in both operating rooms and emergency rooms. Increased utilization is due to the imaging of smaller body parts, the ability to use the machine without a technician, smaller size, and decreased cost. Conflicting studies have shown that the mini C-arm substantially reduces overall radiation exposure to the surgeon but may increase dosage to the hands as they can be in the direct path of the x-ray beam. The use of phantom limbs and cadavers in studies has further been called into question. Though studies have shown minimal risk to surgeons and staff with the use of a mini c-arm, unless in the direct path of the beam, PPE should still be worn by all present to prevent any unnecessary exposure [21–24].

Tuohy et al. [25] reported on 200 consecutive cases performed by four surgeons in which mini c-arm fluoroscopy was used. Dosimeters were worn on the waist under a 0.5 mm lead apron, outside the apron on the pocket over the left chest, and on one of the middle three fingers of the surgeon’s dominant hand. Dosimeters worn under the apron all had minimal (<1 mRem) exposure. Dosimeters on the outside of the apron measured different depths of penetration to simulate penetration depths to the skin, eye, and whole body and showed low rates of scatter. Hand exposure was the greatest, however it was estimated that approximately 7,900 cases would be required to meet the 50,000 mRem annual dose limit for the hands.

Vosbikian et al. [26] found a 10-fold increase in the radiation dosage to the non-dominant hand when using a mini c-arm compared to a large c-arm. When using the mini c-arm the image intensifier was used as a table to mimic common practice, which may have resulted in greater exposure. Regardless, caution was recommended when using the mini c-arm, as the risk to surgeons’ hands may be greater.

### The need for radiation

Another important consideration in radiation safety is the actual need for radiation. Some things to consider when ordering imaging/using fluoroscopy are indications for surgery, indications for special x-ray views, and need for advanced imaging. During fracture fixation we often strive for ‘perfect’ x-rays or a better cosmetic appearance of the fixation leading to unnecessary radiation without adding benefit to the patient. At times imaging studies are done out of fear of medico-legal implications rather than proper indications. This is not to say one should forego proper imaging because of the radiation risk. Going back to the principles of ALARA, the exposure to radiation should be as low as is reasonably achievable, as long the benefits of radiation exposure will outweigh the risks.

### Future directions in intraoperative imaging

The advent of the C-arm changed the way many orthopaedic procedures are performed today. Similarly, advances in technology are now allowing for intraoperative three-dimensional imaging. Three-dimensional imaging can be used to help determine things such as fracture reduction, screw penetrance within a joint, and pedicle screw placement in the spine with greater accuracy and theoretically with less overall radiation exposure. Many of these machines work by taking multiple fluoroscopic images simultaneously from different angles and forming a composite three-dimensional image. As the availability of this technology increases, it is important to study the exposure risks to the operating room staff and patients with these new advancements and also determine their clinical benefits in patients in all fields of orthopaedic surgery.

## Conclusions

While fluoroscopy is a valuable tool that is used daily in orthopaedic surgery, it has its associated risks. A thorough understanding of radiation safety and knowledge of the ALARA principle can help the surgeon obtain quality images while decreasing the amount of harmful radiation exposure. Whenever using fluoroscopy it is important to remember the principle of ‘As Low As Reasonably Achievable’ not only for the patient but for everyone in the operating room. Radiation exposure can be kept as low as reasonably achievable by:

- Using personal protective equipment
- Increasing your distance from the x-ray tube
- Keeping hands out of the direct x-ray beam when possible
- Positioning of the image intensifier as close to the patient as possible
- Using a collimator to decrease the size of the x-ray beam
- Establishing effective communication with the radiology technician

These principles can ensure that the amount of radiation exposure is as low as reasonably achievable in any given scenario.

## Abbreviations

ALARA: As low as reasonably achievable
Gy: Gray
PPE: Personal protective equipment
Rem: Roentgen equivalent man
Sv: Sievert
